# Artificial intelligence-assisted capsule endoscopy for detecting lesions in Crohn’s disease: a systematic review and meta-analysis

**DOI:** 10.3389/frai.2025.1531362

**Published:** 2025-04-01

**Authors:** Yuling Bin, Rumei Peng, Yaqian Lee, Zhijie Lee, Yang Liu

**Affiliations:** ^1^Department of Intensive Care Medicine, Hengyang Central Hospital, Hengyang, China; ^2^Department of Pathology, Changsha Medical University, Changsha, China

**Keywords:** artificial intelligence, capsule endoscopy, Crohn’s disease, convolutional neural network, deep learning

## Abstract

**Background and objectives:**

Crohn’s disease (CD), a complex member of the inflammatory bowel disease spectrum, is characterized by the diversity and skipping distribution of intestinal mucosal lesions, significantly complicating its differential diagnosis with intestinal diseases such as ulcerative colitis and intestinal tuberculosis. With the increasing application of artificial intelligence (AI) in the medical field, its utilization in primary diagnosis has become more widespread. However, there is a lack of systematic evaluation regarding the specific efficacy of AI in identifying CD through capsule endoscopy.

**Methods:**

This study conducted a comprehensive search of PubMed databases, Cochrane, EMBASE, and Web of Science up to May 21, 2024, to collect relevant literature. The Quality Assessment of Diagnostic Accuracy Studies-2 (QUADAS-2) tool was used to rigorously assess the quality of included studies, and detailed information on study characteristics and AI algorithms was extracted. A bivariate mixed-effects model was employed to synthesize and analyze the sensitivity, specificity, and area under the receiver operating characteristic curve (AUC). Additionally, meta-regression and subgroup analyses were conducted to delve into the potential sources of heterogeneity.

**Results:**

Ultimately, eight studies encompassing 11 distinct AI models were included in this meta-analysis. The overall area under the curve (AUC) for AI in identifying CD through capsule endoscopy was 99% (95% CI, 100%-0.00), indicating high diagnostic accuracy. Specifically, the pooled sensitivity was 94% (95% CI, 93–96%), specificity was 97% (95% CI, 95–98%), positive likelihood ratio (PLR) was 32.7 (95% CI, 19.9–53.6), negative likelihood ratio (NLR) was 6% (95% CI, 4–7%), and diagnostic odds ratio (DOR) reached 576 (95% CI, 295–1,127). Meta-regression analysis further revealed that AI algorithm type, study population size, and study design might be key sources of heterogeneity.

**Conclusion:**

This study demonstrates the significant potential of AI technology in assisting endoscopists in detecting and identifying CD patients through capsule endoscopy. However, given the limitations and heterogeneity of current research, more high-quality, large-sample studies are needed to comprehensively and thoroughly evaluate the practical application value of AI in CD diagnosis, thereby promoting its widespread adoption and optimization in clinical practice.

## Introduction

CD is a chronic inflammatory condition that can affect any part of the gastrointestinal tract, from the mouth to the anus, with the terminal ileum and proximal colon being the most commonly involved regions (Torres et al., 2017). It is characterized by a discontinuous, patchy distribution of inflammation (Dolinger et al., 2024). The diagnosis of CD relies on identifying specific findings through endoscopic examination and histological analysis of biopsy samples. Endoscopic features typically include a cobblestone appearance of the mucosa, aphthous ulcers, and skip lesions (Feuerstein and Cheifetz, 2017). Even lesions such as ulcers, fistulas, strictures, and multiple comorbidities may arise during the occurrence and development of CD. These characteristic findings are crucial for distinguishing CD from other inflammatory bowel diseases and for guiding appropriate treatment strategies. So distinguishing CD from intestinal tuberculosis and ulcerative colitis can be challenging.

Capsule endoscopy is a non-invasive diagnostic technique for gastrointestinal diseases, particularly effective in small bowel exploration. It has shown superior performance compared to convolutional endoscopy in terms of CD lesion detection (Kopylov et al., 2017; Tamilarasan et al., 2022). Despite the significant benefits of capsule endoscopy, it faces inherent challenges. These include the ability to observe the entire digestive tract, which leads to a large workload and the inability to focus on a specific area for repeated observation, posing diagnostic difficulties. The large volume of non-targeted images, which can lead to missed diagnoses, even by experienced endoscopists (Brodersen et al., 2024). This problem is even more pronounced for less experienced staff, who often achieve lower detection rates and have less situational awareness. Therefore, enhancing the diagnostic capabilities of less experienced endoscopists in interpreting capsule endoscopy images is highly desirable.

Deep learning (DL), a subset of AI, is primarily based on deep artificial neural networks (Litjens et al., 2017). Convolutional neural network (CNNs), main deep learning algorithm for image analysis, have demonstrated remarkable performance across a wide range of image analysis tasks (Jahagirdar et al., 2023; Xie et al., 2022; Khan et al., 2020). AI is already being utilized in clinical practice (Tao et al., 2025), it has also been successfully applied to gastrointestinal endoscopy images, such as enhancing the detection of polyps (Aziz et al., 2020), tumors (Keshtkar et al., 2023), intestinal tuberculosis (Park et al., 2025), and inflammatory bowel disease(IBD) (Carter et al., 2023) in endoscopy. Thereby improving disease detection rates. AI holds the potential to automatically detect various types of lesions, shorten the reading times of capsule endoscopy and reduce missed diagnoses (Luo et al., 2024). A significant barrier to the adoption of capsule endoscopy for comprehensive gastrointestinal examination can be removed. This meta-analysis aims to synthesize current research on the application of AI in capsule endoscopy for the detection of CD lesions. By integrating data from multiple studies, we evaluate the effectiveness, accuracy, and clinical impact of AI-assisted capsule endoscopy, providing insights into its potential as a diagnostic tool in gastroenterology.

## Method

### Protocol and registration

This systematic review adheres to the rigorous PRISMA (Preferred Reporting Items for Systematic Reviews and Meta-Analyses) guidelines for comprehensive reporting. Our protocol (CRD42024545296) was duly registered with PROSPERO in May 2024, ensuring transparency and reproducibility in our research methodology. All phases of the review-including title and abstract screening, full-text screening, data extraction, assessment of adherence to reporting guidelines, and evaluation of bias and applicability-were independently performed in duplicate by two reviewers. Any disagreements were resolved through discussion with a third independent reviewer.

### Study selection for inclusion

Studies were selected based on inclusion and exclusion criteria that focus on the utilization of AI for the diagnosis of mucosal lesions in CD via capsule endoscopy. Inclusion criteria included peer-reviewed research articles and clinical trials that detail the development or application of AI algorithms specific to capsule endoscopy in CD patients. Exclusion criteria eliminated studies not involving AI, those not using capsule endoscopy, and studies focusing on diseases other than CD.

The search will be conducted across several databases including PubMed, Cochrane, EMBASE, and Web of science using keywords such as “artificial intelligence,” “capsule endoscopy,” and “Crohn’s disease” (Supplementary material). Initial screening will involve reviewing titles and abstracts for relevance. Subsequently, full texts of selected articles will be examined to confirm eligibility. This process will be undertaken independently by two reviewers, with discrepancies resolved by consensus or by consulting a third expert reviewer.

### Data extraction

Data will be extracted using a standardized data collection form developed to capture specific details relevant to the review objectives. Key data points will include: study author(s), publication year, sample size, AI model description (e.g., type of algorithm, training data size), capsule endoscopy findings, and diagnostic accuracy metrics (specificity, sensitivity). Extraction will be conducted by two independent reviewers to ensure accuracy, with a third reviewer available to resolve any discrepancies. Data will be recorded in a structured digital database, which facilitates data management and subsequent analysis.

### Assessment of study characteristics

Eligible studies for inclusion in the review comprised prospective cohort studies, retrospective analyses evaluating the diagnostic accuracy of AI-assisted capsule endoscopy for detecting CD. To qualify, articles needed to report estimates of overall diagnostic accuracy, sensitivity (%), and specificity (%), accompanied by 95% confidence intervals (CIs). There were no restrictions on the size of the studies.

### Risk of bias/quality assessment tool

The Quality Assessment of Diagnostic Accuracy Studies-2 (QUADAS-2) tool was utilized to evaluate the methodological quality of the included articles (Whiting et al., 2011). This tool encompasses four domains: patient selection, index test, reference standard, and flow and timing. Each domain was assessed for high, low, or unclear risk of bias, while the first three domains were also evaluated for high, low, or unclear concerns regarding applicability. The first three areas were also evaluated as applicability issues. Each section is classified as having high, low, or unclear risk of bias. Two investigators independently evaluated the retrieved articles for eligibility, resolving any discrepancies through mutual consensus. Review Manager version 5.3 was employed to create the summary figure for the methodological quality assessment.

### Data synthesis methods

The synthesis of data in this review will be tailored to quantitative analyses, given the technical and clinical variability in the studies. Quantitative synthesis, where data permits, will include a meta-analysis to aggregate diagnostic performance metrics such as sensitivity, specificity, positive predictive value (PPV), negative predictive value (NPV), and AUC from the receiver operating characteristic (ROC) analysis.

### Meta-analysis approach

We used the MIDAS module within STATA (version 18) for statistical analysis. True positives (TP), false negatives (FN), false positives (FP), and true negatives (TN) were constructed to 2×2 contingency tables from artificial intelligence diagnostics of CD patients. The bivariate mixed effects regression model is used for the following indicators: merged sensitivity, specificity, PLR, NLR, DOR, and AUC. Firstly, the heterogeneity of the included studies is evaluated by visually examining the merged comprehensive subject operating characteristic (SROC) curves, and asymmetric shapes indicate significant heterogeneity. Use Spearman correlation analysis to test for heterogeneity caused by threshold effects; Cochran’s Q test and *I*^2^ value test were used to investigate heterogeneity caused by non-threshold effects. If *I*^2^ < 50%, it can be considered that there is low heterogeneity between research results. In this case, a fixed effects model was used for merging; If *I*^2^ ≥ 50%, it can be considered that there is high heterogeneity, and a random effects model is used for merging. Explore the sources of heterogeneity through subgroup analysis and meta regression analysis. Use Deek’s funnel plot to evaluate publication bias, where *p* < 0.1 indicates asymmetric funnel plot. *p* ≤ 0.05 is considered statistically significant. Simultaneously using Fagan’s column chart to evaluate the role and clinical value of AI assisted systems in the diagnosis of CD.

## Result

### Identification of relevant studies

**Figure 1:**
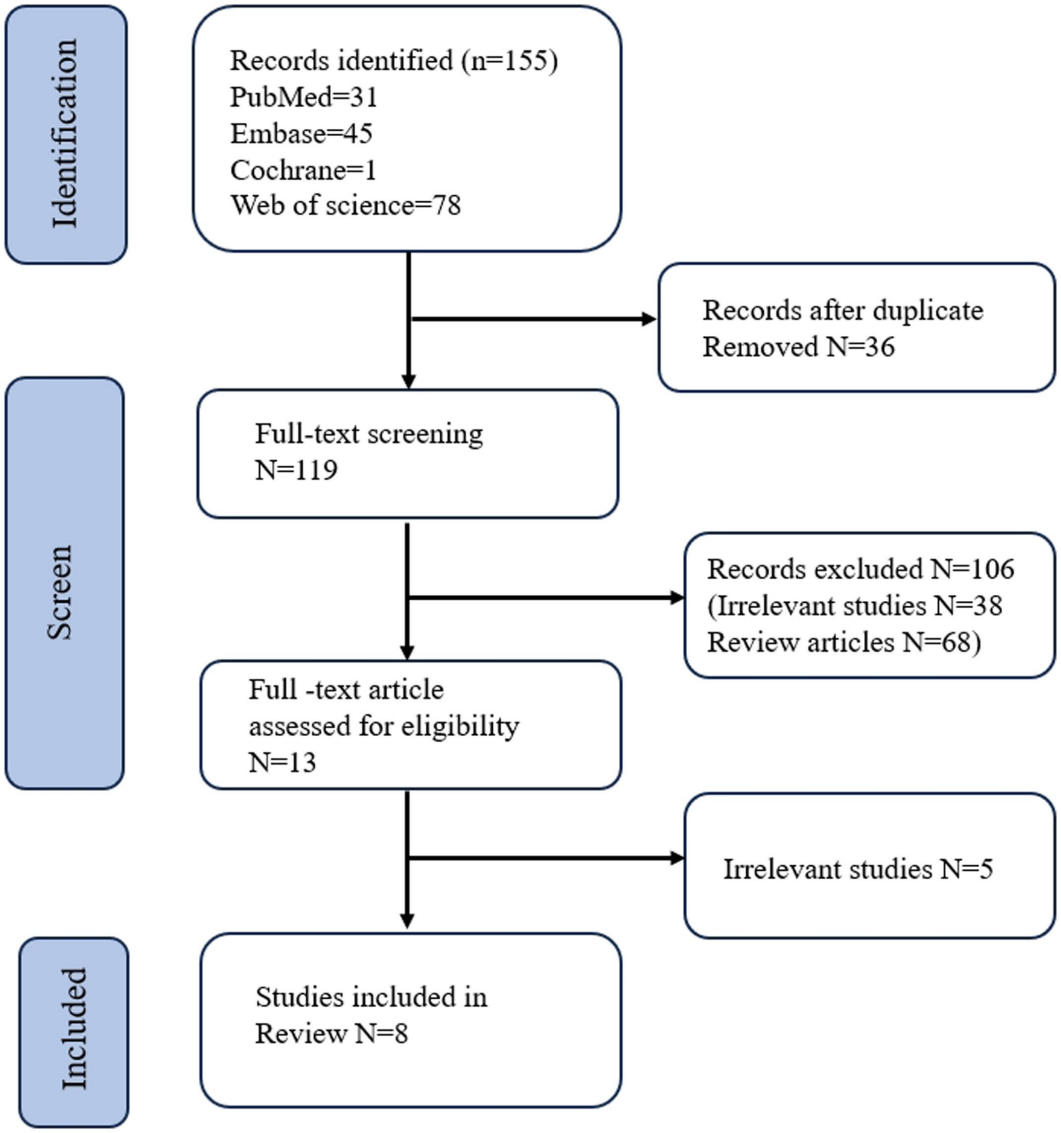
Flow diagram of search methodology and literature selection process.

A total of 155 articles were identified through a search across four electronic databases. Of these, 36 were found to be duplicates and were removed. During the initial screening, which involved a review of titles and abstracts, 106 articles were excluded. The full texts of the remaining 13 articles were thoroughly reviewed. Out of these, five studies were excluded from the final analysis because they did not align with the focus of this systematic review, which is the role of capsule endoscopy in the assessment of CD. Thus, eight studies were included in the final analysis (Figure 1).

### Characteristics of the included studies

All studies assessed the performance of their AI algorithms using an internal validation test dataset, none reported external validation performance. Among the 8 studies diagnosing CD using capsule endoscopy images, a total of 444 patients (353 with CD and 91controls) were identified from 2020 to 2024. Of these studies, 6 were retrospective and 2 were prospective. Most studies developed AI algorithms using deep learning, while three studies employed convolutional neural network algorithms. Almost exclusively, these studies aimed to evaluate the efficacy of the algorithm in CD patients. Klang et al. (2021) selected capsule endoscopy images from 10 CD patients to train a model for detecting intestinal stenosis lesions, while Marin-Santos et al. (2023) utilized capsule endoscopy images from 38 CD patients to identify various lesions in the intestinal mucosa. An exception to this trend is the study conducted by Brodersen et al. (2024), which notably did not exclude patients with ulcerative colitis and cancer. The number of participants in each study displayed a wide range, varying from a minimum of 10 to a maximum of 133 individuals. Moreover, a limitation across these retrospective studies was the absence of reporting the average age and gender of the participants. Most studies established the AI algorithm based on the deep learning. The included studies could be categorized by analysis based on design, AI algorithm, the number of enrolled patients. These characteristics were evaluated as potential sources of heterogeneity through the subgroup analysis and meta-regression. Detailed characteristics of the studies are presented in Table 1.

**Table 1:** Summary of studies in the literature review that applied AI techniques for capsule endoscopy image analysis.

Study	Year	Study type	Type of AI		Comparator	Dataset size	Application	TP	FP	FN	TN
Klang et al. (2020)	2020	Retrospective	CNN	(DL Fold1)	2 gastroenterology fellows	17,640 images of 49 patients from department of gastroenterology at Sheba Medical Center	Ulceration	6,837	195	554	10,054
				(DL Fold2)				6,933	338	458	9,911
				(DL Fold3)				7,177	410	214	9,839
				(DL Fold4)				6,999	256	392	9,993
				(DL Fold5)				7,154	348	237	9,901
Vallée et al. (2020)	2020	Retrospective	Neural networks	(ResNet 34)	3 independent experts	3,498 images of 63 patients from the Nantes University Hospital	Ulceration, Stenosis, Edema, Erythema	1,506	89	124	1,645
				(ResNext)				1,509	112	121	1,622
				(VGGNet 19)				1,491	109	139	1,625
				(VGGNet 16)				1,493	115	137	1,619
De Maissin et al. (2021)	2021	Retrospective	Neural networks	CRANN	1 gastroenterology fellow and 3experts.	3,498 images of 63 patients from CrohnIPI dataset	Erythema, Edema, Ulceration, Stenosis	1,241	76	119	2048
				ResNet 34				1,241	81	119	2043
				VGGNet 16				1,206	89	154	2035
				VGGNet 19				1,199	101	161	2023
Klang et al. (2021)	2021	Retrospective	Neural Network		A capsule expert	27,89 images of 10 patients	Strictures	815	590	71	4,772
Majtner et al. (2021)	2022	Prospective	DL (ResNet-50)	Small bowel	3 gastroenterologists	774 images from 38 CD patients in 3 managing centers	Hyperplasia, Ulceration, Fissure	359	0	15	620
				Colon				174	0	6	302
				Overall				533	0	21	922
Ferreira et al. (2022)	2022	Retrospective	CNN (Xception model)		3 gastroenterologists	2,449 images of 59 patients from a multicenter study	Ulceration, Erosions	5,194	192	106	18,998
Marin-Santos et al. (2023)	2023	Retrospective	CNN		2 digestive specialists	15,972 images of 31 CD patients from Juan Ramón Jiménez hospital	Ulceration, Bleeding, Erythema, Nodules, Erosion, etc.	1,581	59	16	1,538
Brodersen et al. (2024)	2024	Prospective	DL (AXARO framework)		2 GI-Specialists	86,129 images from 131 CD patients from Southern Denmark managing	Vascular abnormalities, Inflammatory, Ulcerations, bleeding, Polypus	49	6	2	74
								48	8	4	71

AI, Artificial Intelligence; CNN, Convolutional neural network; DL, Deep learning; TP, True positives; FP, false positives; FN, false negatives; TN, true negatives.

### Quality evaluation of included literature

Regarding the QUADAS-2 tool, the quality assessment results of included studies are summarized in Table 2. Among the 8 studies included in the final analysis, 2 demonstrated a low risk of bias, 6 study at unclear risk. Bias in the included studies primarily stems from the domains of patient selection, index test, flow and timing.

**Table 2:** Quality Assessment of Diagnostic Accuracy Studies-2 risk for the assessment of the methodological qualities.

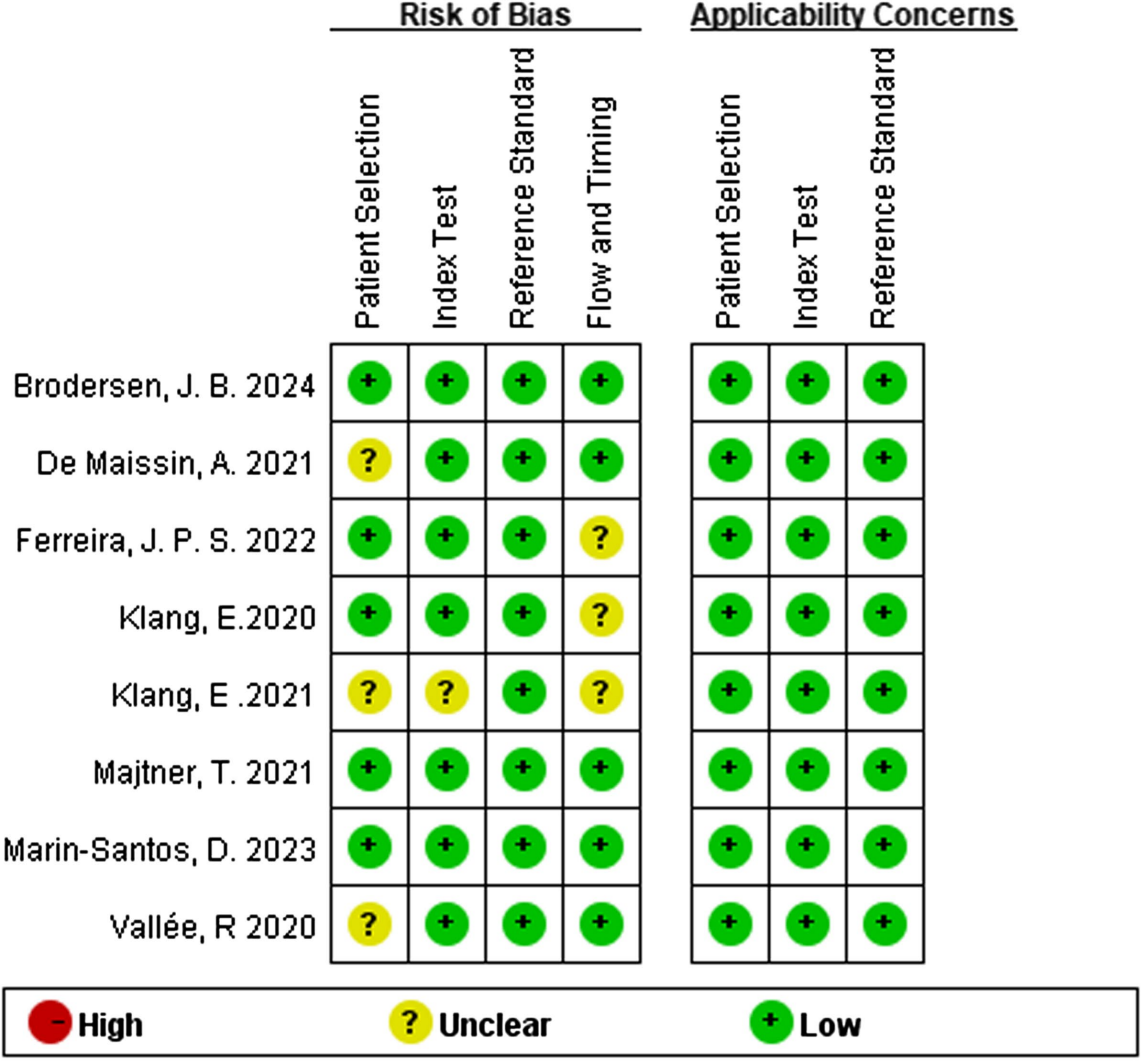

(+) denotes low risk of bias, (?) denotes unclear risk of bias, (−) denotes high risk of bias.

### Diagnostic test accuracy of artificial intelligence for the Crohn’s disease

**Figure 2:**
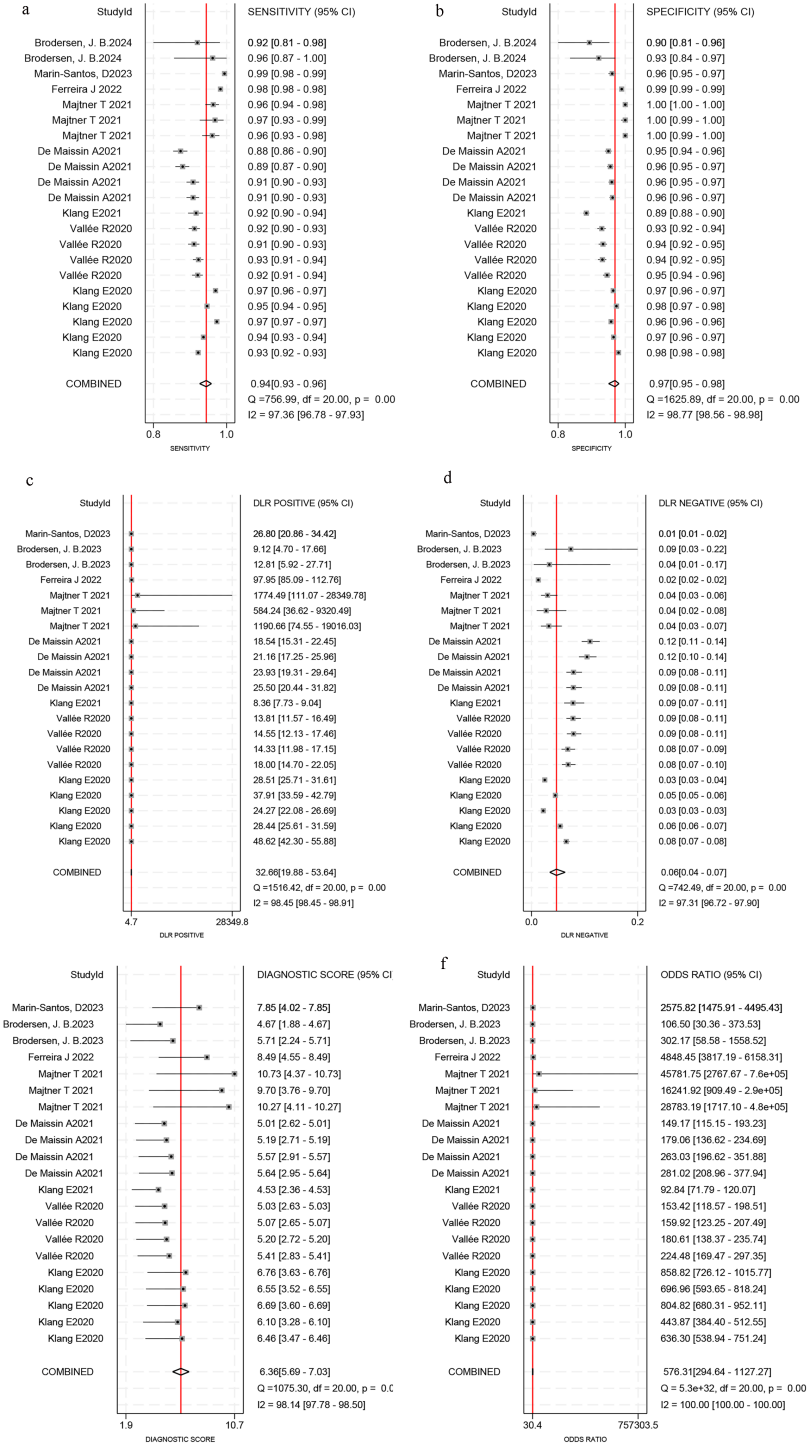
Forest plots reveal sensitivity, specificity, positive likelihood ratio (PLR), negative likelihood ratio (NLR), diagnostic score and diagnostic odds ratio (DOR) estimates of Artificial Intelligence for Crohn’s disease in capsule endoscopy images. Sensitivity **(a)**, specificity **(b)**, PLR**(c)**, NLR **(d)**, diagnostic score **(e)**, and DOR **(f)**.

**Figure 3:**
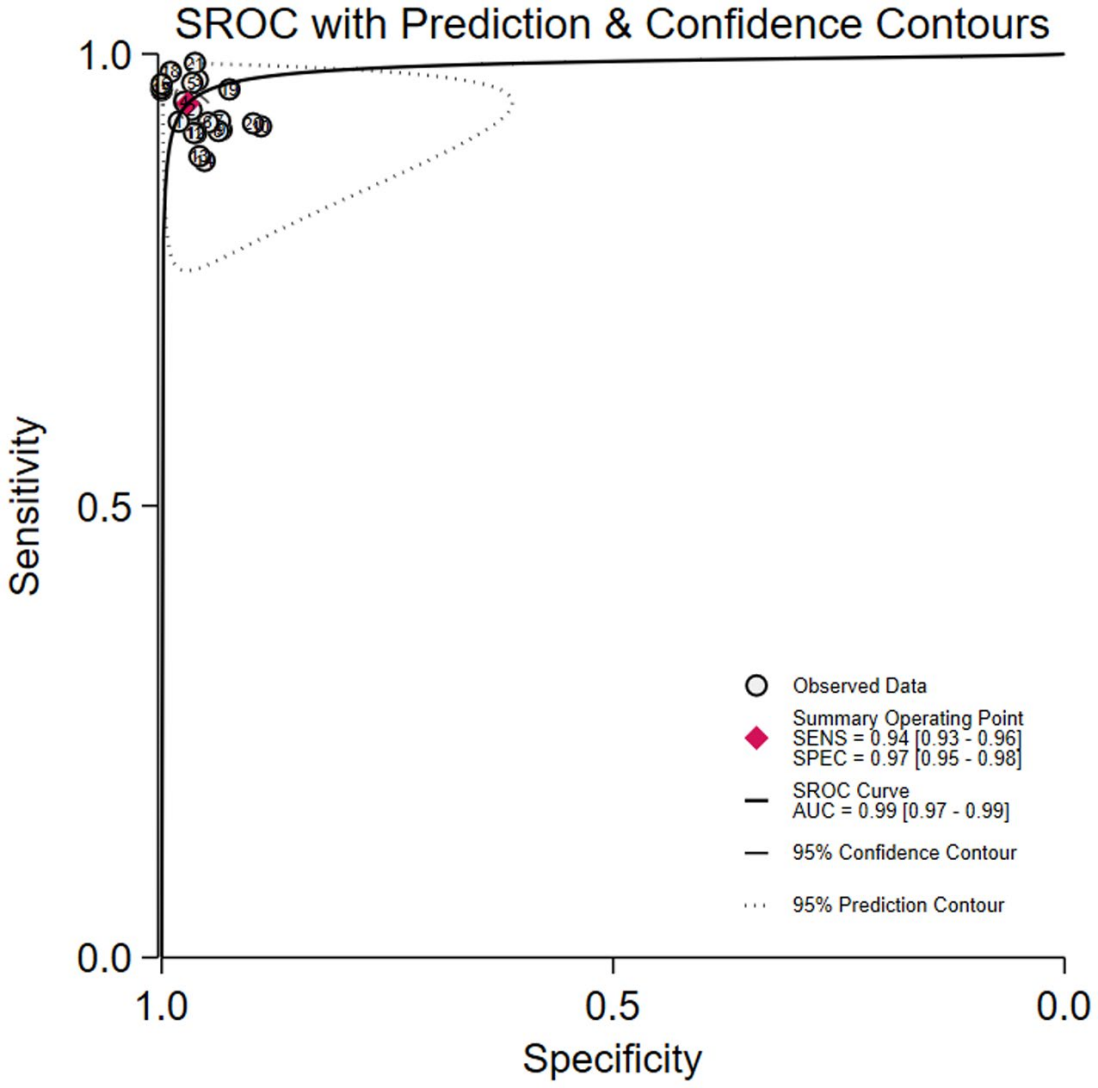
Summary receiver operating characteristic (SROC) curve with 95% confidence and prediction regions for Crohn’s disease detection in capsule endoscopy.

**Figure 4:**
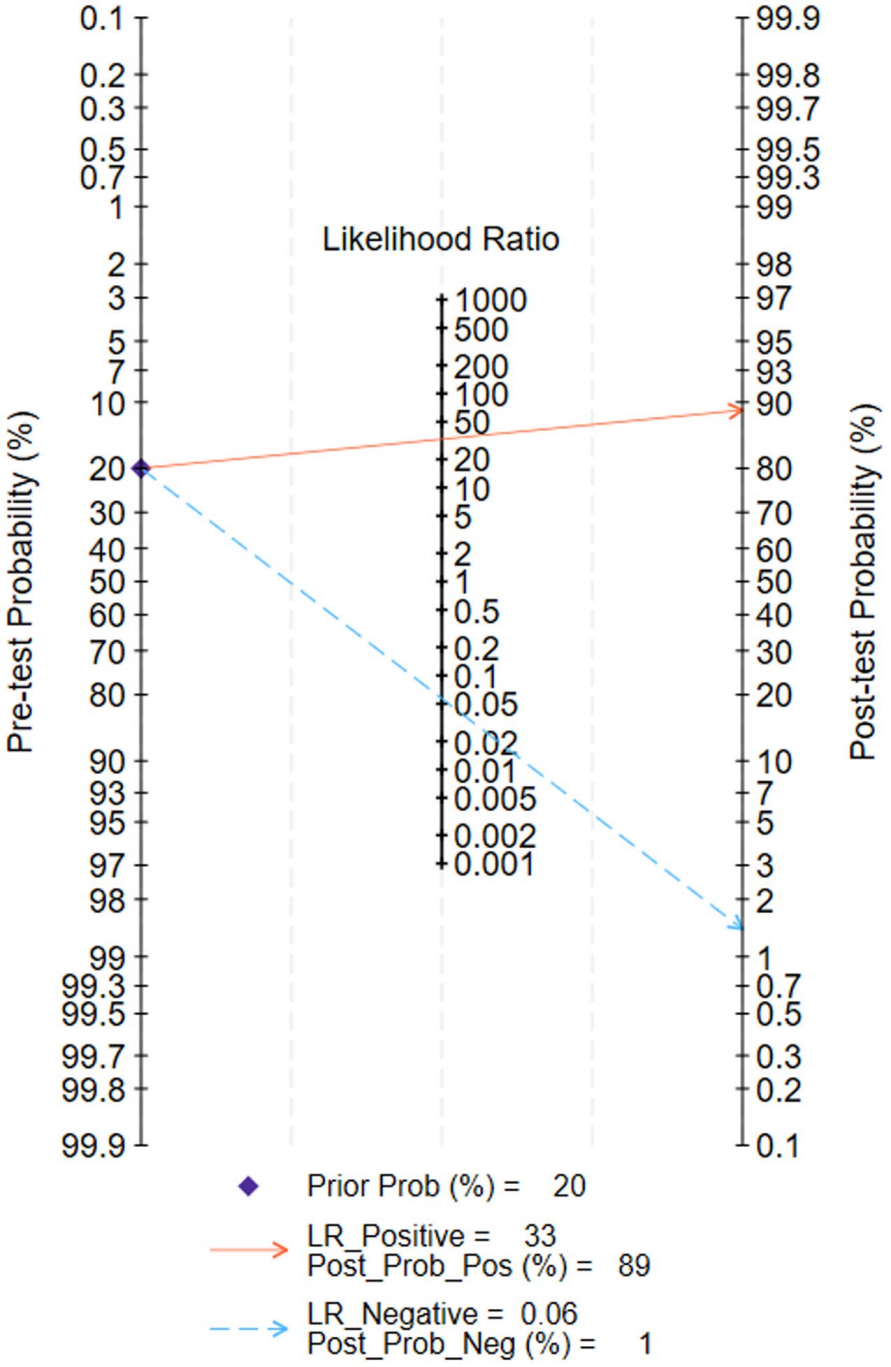
Fagan nomogram for the diagnosis of Crohn’s disease in capsule endoscopy images.

Among the 8 studies of patient-based analysis, the sensitivity (Figure 2a), specificity (Figure 2b), PLR (Figure 2c), NLR (Figure 2d), diagnostic score (Figure 2e), and DOR (Figure 2f) of AI for CD were 0.94 (95% CI, 0.93-0.96), 0.97 (95% CI, 0.95-0.98), 32.7 (95% CI, 19.9-53.6), 0.06 (95% CI, 0.04-0.07), 6.36 (95% CI, 5.69-7.03), and 576 (95% CI, 295-1127), respectively, in Figure 2. The summary receiver operating characteristic (SROC) curve for the included studies (*n* = 8) was presented in Figure 3, where the calculated area under the curve (AUC) was 0.99 (95% CI, 0.97-0.99). To investigate the clinical utility of AI, a Fagan nomogram was generated. As shown in Figure 4, with a pretest probability of 20%, the post-test probability for a positive result was 89%. Given a negative test result, the negative likelihood ratio of 0.06 reduced the posterior probability to 1%.

### Exploring heterogeneity with meta-regression

**Figure 5:**
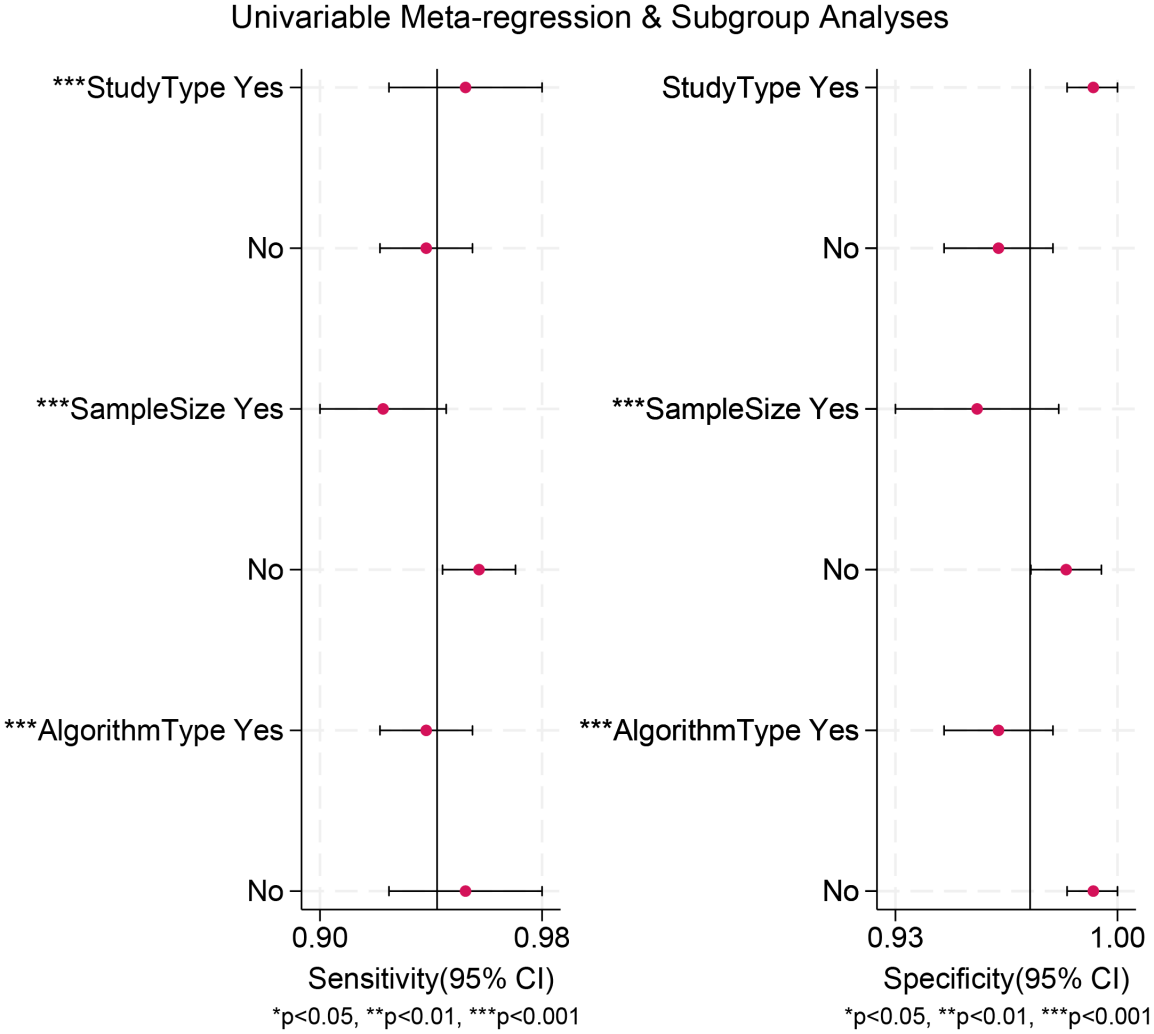
Meta-regression analysis identifying sources of heterogeneity in the diagnostic test accuracy meta-analysis.

The meta-analysis of AI-assisted capsule endoscopy for CD lesion detection revealed significant heterogeneity. To explore the sources of this heterogeneity, we performed meta-regression and subgroup analyses. Results demonstrated that heterogeneity in sensitivity was significantly influenced by design type, sample size, and algorithm type; whereas heterogeneity in specificity was primarily attributed to sample size and algorithm type (Table 3 and Figure 5).

**Table 3:** The subgroup analysis of AI- assisted capsule endoscopy for detecting CD lesion.

Subgroup	Study Type	N	Sensitivity	p1 value	Specificity	p2 value
Design Type	Prospective	5	0.96 (0.93 - 0.9)	0.00	0.99 (0.98-1.00)	0.67
	Retrospective	16	0.94 (0.92- 0.96)	.	0.96 (0.94 - 0.98)	.
Sample Size	Number>50	11	0.93 (0.90 - 0.95)	0.00	0.95 (0.93-0.98)	0.00
	Number<50	10	0.96 (0.95 - 0.97)	.	0.98 (0.97 - 0.99)	.
Algorithm Type	Deep Learning	16	0.94 (0.92 - 0.96)	0.00	0.96 (0.94 - 0.98)	0.00
	Other Algorithm	5	0.96 (0.93 - 0.98)	.	0.99 (0.98 - 1.00)	.

N, Number of included studies.

### Publication bias

**Figure 6:**
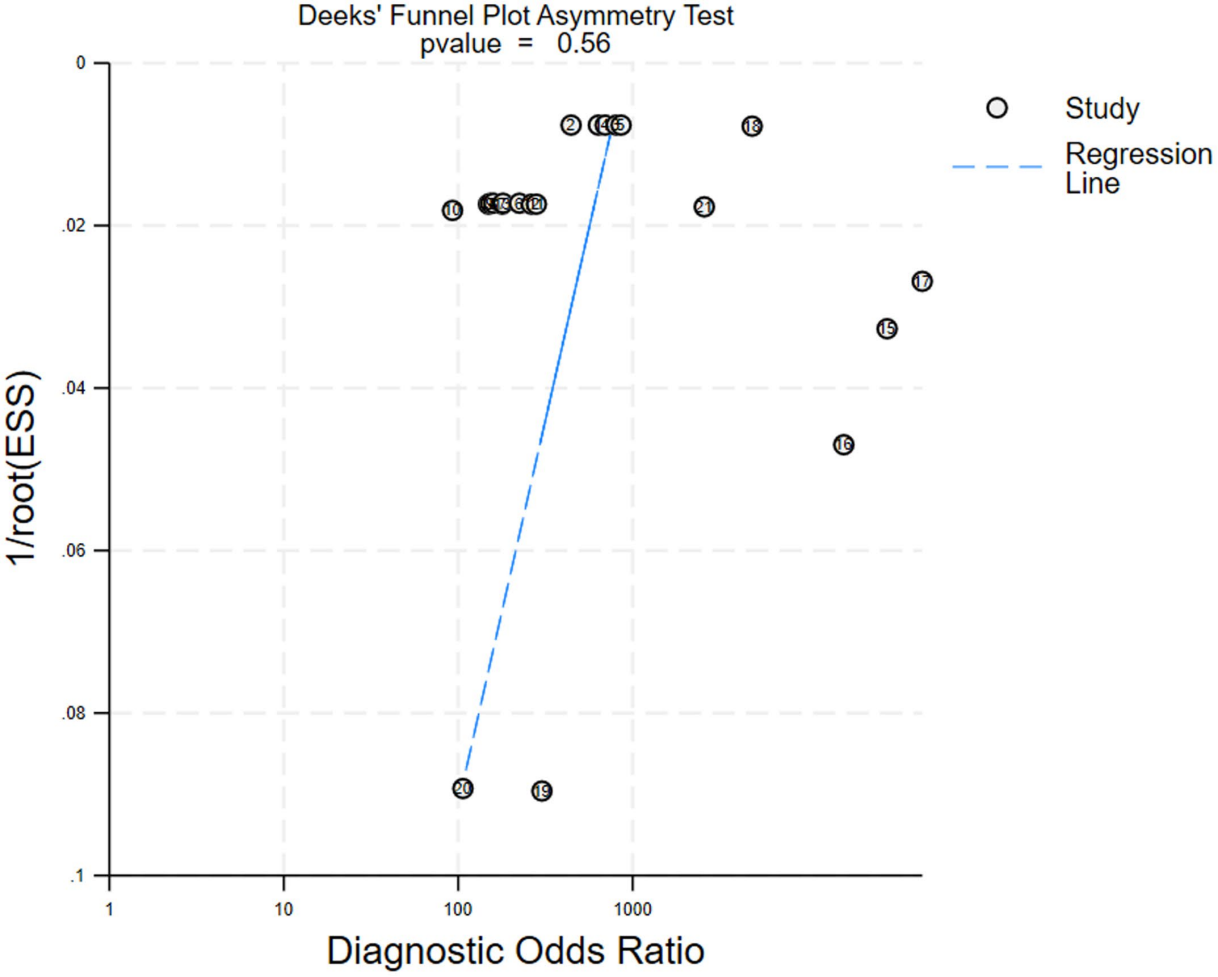
Deek’ funnel plot for the studies of assessing publication bias (*p* = 0.56), indicating no significant publication bias. Each dot represents an individual study.

We performed a publication bias analysis for the included studies. No significant bias was observed in diagnostic Crohn’s disease efficacy of AI via capsule endoscopic images. Figure 6 shows the Deek’ funnel plot indicated no evidence of publication bias (*p* = 0.56).

## Discussion

### Principal findings

CD is an autoimmune disorder that affects the entire digestive tract and can progress rapidly (Chapman and Satsangi, 2024). Capsule endoscopy offers significant advantages for diagnosing CD, as it minimizes the need for multiple invasive procedures and reduces patient discomfort (Marquès Camí et al., 2020). However, capsule endoscopy produces a large volume of images, some of which may be blurry or irrelevant. Moreover, gastrointestinal mucosal lesions of CD include various manifestations such as edema, ulcers, fistulas, stenosis, etc., all of which contribute to the complexity and challenge of making an accurate diagnosis (Deng et al., 2024). Especially for inexperienced clinicians in primary hospitals, differentiating CD, UC and non-IBD colitis has remained a dilemma.

Traditionally, endoscopy diagnosis is operator-dependent and subjective, recent studies have demonstrated the substantial potential of AI in medical diagnostics (Khan et al., 2020; Mitsala et al., 2021; Huang et al., 2022). AI-assisted endoscopy can provide a valuable second opinion, potentially reducing operator dependency (Parkash et al., 2022). This approach, characterized as computer-aided diagnosis, significantly improves the diagnostic accuracy of endoscopy. Endoscopist combined with AI analysis will enhance the probability of uncovering crucial gastrointestinal mucosal lesions under endoscopy, thereby advancing the accuracy and effectiveness of diagnostic assessments. Recently, deep learning, as the subset of AI, grown dramatically with a high overall coverage in medical research. Based on the detailed description of Table 1, it is evident that the deep learning algorithm undergoes constantly updates. Thousands of computational iterations, these algorithms can “learn” the unique features of images or data with a given classification, then emulate human problem-solving and identify specific capsule endoscopy images of CD. Klang et al. (2020) presented the results of 5 experiments performed on randomly split images (80% training, 20% testing), ultimately enhancing classification accuracy through the integration of these data. Throughout the validation phase, they demonstrated an enhanced performance, achieving accuracies ranging from 95.4 to 96.7%. Vallée et al. (2020) and De Maissin et al. (2021) employed a multifaceted strategy incorporating a diverse array of deep learning models to secure a thorough assessment of the characteristics of histological images and endoscopic data, yielding an overall F1 score of 91.46% with the VGG16 system. Marin-Santos et al. (2023) demonstrated high recognition accuracy utilizing the convolutional neural network system, exhibiting substantial precision in test phase and achieving a 95–99% sensitivity range.

This article provides a comprehensive review of the diverse applications of AI in diagnosing CD patients through the analysis of capsule endoscopy images. Deep learning exhibits high diagnostic accuracy for CD patients undergoing capsule endoscopy, particularly when utilizing convolutional neural networks. The applications of AI are not only in diagnose CD, but also in prognosis and predicting treatment response. It is essential for managing diseases, mitigating the risk of prolonged complications, and enhancing the quality of life for CD patients. A significant finding of this study is the robustness of the AI algorithm’s diagnostic performance, while studies involving a large patient population and high methodological quality demonstrated higher diagnostic performance, the difference was not substantial.

### Limitations

Despite employing a comprehensive and thorough search strategy, several limitations must be acknowledged regarding both the evidence and the review itself. We were able to identify only 8 eligible studies. Any of these studies presented an unclear risk of bias due to their design. But most studies either had small sample sizes or included only patients with CD, which may have compromised the precision of the effect estimates. The studies included in the meta-analysis lack external data validation, which compromises the reliability of their findings. Most studies utilize deep learning as the AI algorithm, with only 3 studies employing convolutional neural network.

Compared to previous AI-assisted studies in endoscopy, those applications are predominantly utilized to differentiate polyps, tumors, peptic ulcers, and IBD. This review has demonstrated that the majority of the included studies are capable of detecting multiple abnormalities in patients with CD. However, differentiating between IBD subtypes remains a notable gap, particularly due to their complexity and the significant variability observed among individual patients. AI-assisted colonoscopy applications specifically designed for UC patients have been introduced exclusively in Japan. Due to the requirement of specialized endoscopes, this software has not been widely adopted in clinical practice (Ogata et al., 2025). Additionally, it suffers from multiple biases, particularly the unequal distribution of CD patients among the participants, the absence of randomization in selecting subjects, the utilization of datasets from different resources, and the use of more uneven image resolution, all of which restrict the generalizability of the findings. Although these findings employing a multimodal imaging technique which facilitated a more thorough evaluation of histological characteristics, AI applications for assessing architectural modifications and inflammatory infiltrates were less impressive, further advancements are necessary to enhance the accuracy and dependability in identification. This meta-analysis also presents the results of an advanced AI-assisted diagnostic system, but the studies included were unable to detect and analyze CD activity, and estimate the corresponding endoscopic activity. To enhance diagnostic capabilities, AI must be trained and rigorously validated using vast amounts of data, allowing it to learn the intricacies of these diverse manifestations and ultimately improve its diagnostic accuracy. Therefore, more prospective studies focusing on the application of AI in clinical practice, particularly in diagnostic equipment, is essential.

## Conclusion

AI algorithms have demonstrated high accuracy in identifying various gastrointestinal pathologies, including polyps, neoplasms and peptic ulcer infected with *helicobacter pylori*. This meta-analysis has presented the application of AI to enhance the primary diagnosis of CD patients in capsule endoscopy images, by facilitating the identification and classification of specific patterns and lesions in histological images. It aimed to synthesize the existing evidence on the diagnostic accuracy of AI models for identifying lesions associated with CD in capsule endoscopy, and attempted to address crucial gaps in the current literature. Our study indicated a sensitivity of 94% and a specificity of 97% when utilizing AI for diagnosing CD in capsule endoscopy images, presenting the potential to revolutionize its treatment and diagnosis. However, heterogeneity and small sample sizes compromise the quality and validity of these findings. The currently lack sufficient data to accurately identify intestinal mucosal lesions such as ulcers, stenosis, and bleeding. Further research, including rigorous patient’s selection and randomized clinical trials, is needed to fully assess the effectiveness of AI in capsule endoscopy. Additionally, the possibility of missing potential lesions in AI-assisted analysis of capsule endoscopy results cannot be dismissed, despite the potential benefit of shortened reading times. Overall, it offers a comprehensive overview of these models, serving as a foundation for future research in this field. Such studies could help promote more individualized and flexible screening options for suspected CD patients, to minimize invasive examination.
