# Supplementary material for: Artificial intelligence-assisted capsule endoscopy for detecting lesions in Crohn’s disease: a systematic review and meta-analysis
Source: Front Artif Intell. 2025 Apr 1;8:1531362. doi: 10.3389/frai.2025.1531362 (PMC11996797; doi:10.3389/frai.2025.1531362)
Supplement: Supplementary file 1 [file Data_Sheet_1.docx]

**Appendix**: Searching strategy to find the relevant articles.

**Pubmed:**

1."artificial intelligence"[tiab] OR "AI"[tiab] OR "deep learning"[tiab] OR "machine learning"[tiab] OR "computer aided"[tiab] OR "neural network"[tiab] OR "CNN"[tiab]

2. ("Crohn Disease"[Mesh] or Crohn disease[tw] or Crohn's Enteritis[tw] or Regional Enteritis[tw] or Crohn's Disease[tw] or Crohns Disease[tw] or Inflammatory Bowel Disease 1[tw] or Ileocolitis[tw] or Granulomatous Colitis[tw] or Terminal Ileitis[tw] or Regional Ileitides[tw] or Regional Ileitis[tw])

3. ((((capsule endoscop*[MeSH Terms]) OR (capsule endoscop*[Title/Abstract])) OR (capsule colono*[Title/Abstract])) OR (pan-*endoscop*[Title/Abstract])) OR (wireless capsule endoscop*[Title/Abstract])

4. (("artificial intelligence"[tiab] OR "AI"[tiab] OR "deep learning"[tiab] OR "machine learning"[tiab] OR "computer aided"[tiab] OR "neural network"[tiab] OR "CNN"[tiab]) AND (("Crohn Disease"[Mesh] or Crohn disease[tw] or Crohn's Enteritis[tw] or Regional Enteritis[tw] or Crohn's Disease[tw] or Crohns Disease[tw] or Inflammatory Bowel Disease 1[tw] or Ileocolitis[tw] or Granulomatous Colitis[tw] or Terminal Ileitis[tw] or Regional Ileitides[tw] or Regional Ileitis[tw]))) AND (((((capsule endoscop*[MeSH Terms]) OR (capsule endoscop*[Title/Abstract])) OR (capsule colono*[Title/Abstract])) OR (pan-* endoscop*[Title/Abstract])) OR (wireless capsule endoscop*[Title/Abstract]))

**Embase**

1. 'artificial intelligence': ab,ti,kw OR 'AI':ab,ti,kw OR 'deep learning':ab,ti,kw OR 'machine learing':ab,ti,kw OR 'computer aided':ab,ti,kw OR 'neural netwokr':ab,ti,kw OR 'CNN'
2. 'crohn disease':ab,ti,kw OR 'crohn* enteritis':ab,ti,kw OR 'regional enteritis':ab,ti,kw OR 'inflammatory bowel disease':ab,ti,kw OR 'ileocolitis':ab,ti,kw OR 'granulomatous colitis':ab,ti,kw OR 'terminal ileitis':ab,ti,kw OR 'regional ileitides':ab,ti,kw OR 'regional ileitis':ab,ti,kw
3. 'capsule endoscopy'/exp OR 'capsule endoscopy' OR 'capsule endoscop*': ab,ti,kw OR 'capsule colono*':ab,ti,kw OR 'wce*':ab,ti,kw OR 'wireless capsule endoscop*':ab,ti,kw

**Cochrane**

#1 MeSH descriptor: [Artificial Intelligence] explode all trees

#2 (artificial intelligence):ab,ti,kw OR (Deep Learning):ab,ti,kw OR (machine learning):ab,ti,kw OR (computer aided):ab,ti,kw OR (hierarchical learning):ab,ti,kw OR (computational intelligence):ab,ti,kw OR (machine intelligence):ab,ti,kw OR (computer reasoning):ab,ti,kw OR (classification algorithm):ab,ti,kw OR (feed-forward neural network):ab,ti,kw OR (convolutional neural network):ab,ti,kw OR (convolutional network):ab,ti,kw OR (neural network):ab,ti,kw

#3 (Crohn* disease): ab,ti,kw OR (Crohn*):ab,ti,kw

#4 MeSH descriptor: [Crohn Disease] explode all trees

#5 MeSH descriptor: [Capsule Endoscopy] explode all trees

#6 (capsule endoscop*): ab,ti,kw OR (capsule colono*):ab,ti,kw OR (pan-* endoscop*):ab,ti,kw OR (WCE*):ab,ti,kw OR (wireless capsule endoscop*):ab,ti,kw

#7 (#1 OR #2) AND (#3 OR #4) AND (#5 OR #6)

**Web of science**

((TS = (artificial intelligence or Deep Learning or machine learning or computer aided or hierarchical learning or computational intelligence or machine intelligence or computer reasoning or classification algorithm or feed-forward neural network or convolutional neural network or convolutional network or neural network)) AND TS = (Crohn* disease or Crohn*)) AND TS = (capsule endoscop* or capsule colono* or pan-*endoscop* or WCE* or wireless capsule endoscop*)
